# Extensive proteome and functional genomic profiling of variability between genetically identical human B-lymphoblastoid cells

**DOI:** 10.1038/s41597-022-01871-9

**Published:** 2022-12-10

**Authors:** Miklós Laczik, Edina Erdős, Lilla Ozgyin, Zsuzsanna Hevessy, Éva Csősz, Gergő Kalló, Tibor Nagy, Endre Barta, Szilárd Póliska, István Szatmári, Bálint László Bálint

**Affiliations:** 1 Genomic Medicine and Bioinformatic Core Facility, Department of Biochemistry and Molecular Biology, Faculty of Medicine, University of Debrecen, Debrecen, Egyetem tér 1., H-4032 Hungary; 2 Department of Laboratory Medicine, Faculty of Medicine, University of Debrecen, Debrecen, Egyetem tér 1., H-4032 Hungary; 3 Proteomics Core Facility, Department of Biochemistry and Molecular Biology, Faculty of Medicine, University of Debrecen, Debrecen, Egyetem tér 1., H-4032 Hungary; 4 Department of Genetics and Genomics, Institute of Genetics and Biotechnology, Hungarian University of Agriculture and Life Sciences, Szent-Györgyi Albert út 4, Gödöllő, H-2100 Hungary; 5 Faculty of Pharmacy, University of Debrecen, Debrecen, Egyetem tér 1., H-4032 Hungary; 6 Department of Bioinformatics, Semmelweis University, Budapest, Tűzoltó utca 7-9., H-1094 Hungary

**Keywords:** Transcriptomics, Proteomics

## Abstract

In life-science research isogenic B-lymphoblastoid cell lines (LCLs) are widely known and preferred for their genetic stability – they are often used for studying mutations for example, where genetic stability is crucial. We have shown previously that phenotypic variability can be observed in isogenic B-lymphoblastoid cell lines. Isogenic LCLs present well-defined phenotypic differences on various levels, for example on the gene expression level or the chromatin level. Based on our investigations, the phenotypic variability of the isogenic LCLs is accompanied by certain genetic variation too. We have developed a compendium of LCL datasets that present the phenotypic and genetic variability of five isogenic LCLs from a multiomic perspective. In this paper, we present additional datasets generated with Next Generation Sequencing techniques to provide genomic and transcriptomic profiles (WGS, RNA-seq, single cell RNA-seq), protein-DNA interactions (ChIP-seq), together with mass spectrometry and flow cytometry datasets to monitor the changes in the proteome. We are sharing these datasets with the scientific community according to the FAIR principles for further investigations.

## Related content

Ozgyin, L., Horváth, A., Hevessy, Z., Bálint, B.: Extensive epigenetic and transcriptomic variability between genetically identical human B-lymphoblastoid cells with implications in pharmacogenomics research^1^. Sci Rep. 9 1–16, 2019. 10.1038/s41598-019-40897-9

## Background & Summary

Genotyped human B-lymphoblastoid cell lines (LCLs) are widely used in various biomedical studies^2–10^. These premature B-cells are transformed by the Epstein-Barr virus^11^, so that it has a minimal effect on the genotype or the expression profile. Although viral genes are present in episomes, various studies show that their expression is low and the general expression profile does not show a high correlation with viral patterns, even though it can vary from cell to cell^12–15^.

The B-lymphoblastoid cells are usually generated from the peripheral blood of a subject. One of the advantages and acclaimed features of LCLs is their genetic stability that makes them widely used in genetic studies and various investigations, such as studies of induced mutations or drug/carcinogen responses, where it is crucial that the results should not be biased by variations in the genomic sequence. Several studies have been conducted on LCLs in this context^16–19^.

In our previous publication^1^ we presented evidence that even isogenic B-lymphoblastoid cells show phenotypic differences. These changes could not be explained by the experimental conditions and were documented by performing RNA-Seq based global gene expression analysis and ChIP-Seq based chromatin profiling. While these cells were claimed isogenic at that time, we had no experimental data about the possible genetic differences of these cells. Our hypothesis was that although these cells are mostly stable, a certain portion of them can exhibit genomic mutations, which in turn leads to alterations in regulatory processes and subsequently gene expression. If this is true, the extent of this systemic bias must be measured and accounted for in future LCL studies, and it might make us reconsider some of the conclusions drawn from previous LCL experiments^1^.

To gain deep insight into the variations in human LCLs we sequenced the whole genome using Next Generation Sequencing technology (WGS) and identified genetic variations within these isogenic LCL cell lines that were derived from the same person. The WGS datasets are complemented with ChIP-seq data that allow us to profile histone modification loci, together with gene expression by RNA-seq datasets that describe the global gene expression landscape of the investigated cell lines. For selected samples we performed single cell RNA-seq (scRNA-seq) that reveals transcription variability on a single cell level. Note that, according to our knowledge, this is the first time that scRNA-seq is beeing published from LCL samples. The actual changes in protein levels were measured by mass spectrometry and flow cytometry.

We collected the datasets of B-lymphoblastoid cell lines that our research group had generated before^1^, and we performed additional experiments that could provide further insight into the variability of LCL cells. With all these datasets we strive to prove that despite their relative stability, the genotyped human B-lymphoblastoid cell lines can show various degrees of variation that can influence the results of a scientific experiment.

Further studies are needed to clarify if the observed phenotypic changes are results of stochastic events or of the minor genetic changes identified.

We aim to provide a compendium of LCL data^20–26^ and share it according to the FAIR principles^27^ so the scientific community can study the various aspects of genomic and epigenomic features of the B-lymphoblastoid cell lines and understand their variations.

We believe that good data management and data stewardship are crucial in scientific research: making a discovery from your data should not be the end of your work as a researcher, it is also important to provide long term community access to the data and data processing protocols in a manner that the data may be reproduced, evaluated, reused and integrated in future research. The FAIR principles guide the scientist along that road, and they have been widely adopted since their inception; see FAIR sharing for the community and an up-to-date collection of databases, standards and policies^28^.

## Methods

**Fig. 1:**
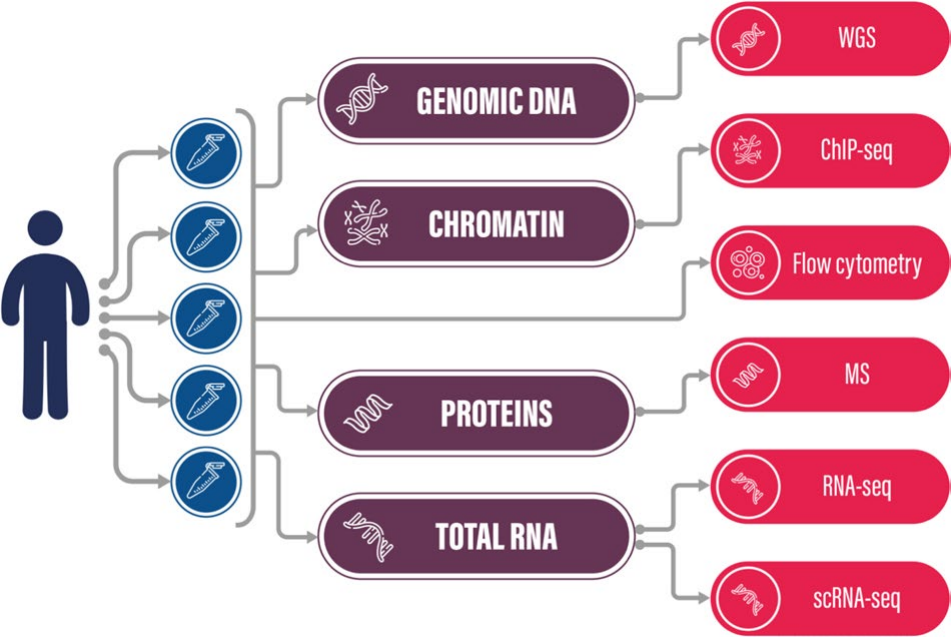
A general overview of the experiment design and data types.

The methods described here represent an extended methodology of our recently published and related publication^1^. Descriptions for cell culture, flow cytometry, and the wet-lab processing of H3K27ac ChIP-Seq and RNA-Seq experiments can also be found there, we highlighted that article as Related content. The sampling scheme and the various experiments conducted on the cell lines are visualized in Fig. 1.

### Whole genome sequencing

#### Sample preparation and sequencing

Human B-lymphoblastoid cell lines derived from five different tubes of anticoagulated peripheral blood, drawn from the same 26-year-old CEPH/UTAH male (GM22647, GM22648, GM22649, GM22650 and GM22651), were obtained from Coriell Cell Repositories. Cells were cultured in RPMI-1640 supplemented with 15 v/v% heat-inactivated FCS, 2 mM L-glutamine and 1 v/v% penicillin-streptomycin. Genomic DNA was isolated using a High Pure PCR Template Preparation Kit (Roche Life Science, cat. 11796828001). Library preparation was performed by Novogene Co., Ltd. from 1 microgram of DNA per sample based on the NEBNext DNA Library Prep Kit following the manufacturer’s recommendations. The genomic DNA is randomly fragmented to a size of 350 bp by ultrasound shearing, then DNA fragments were end polished, A-tailed, and ligated with the NEBNext adapter for Illumina sequencing, and further PCR enriched by P5 and indexed P7 oligos. The PCR products were purified (AMPure XP system) and resulted libraries were analysed for size distribution by Agilent 2100 Bioanalyzer and quantified using real-time PCR. The qualified libraries were sequenced by Illumina NovaSeq. 6000 sequencers with 150 bases long paired-end reads, after pooling according to its effective concentration and expected data volume, using multiple lanes per volume to mitigate batch effect.

#### Data analysis

**Fig. 2:**
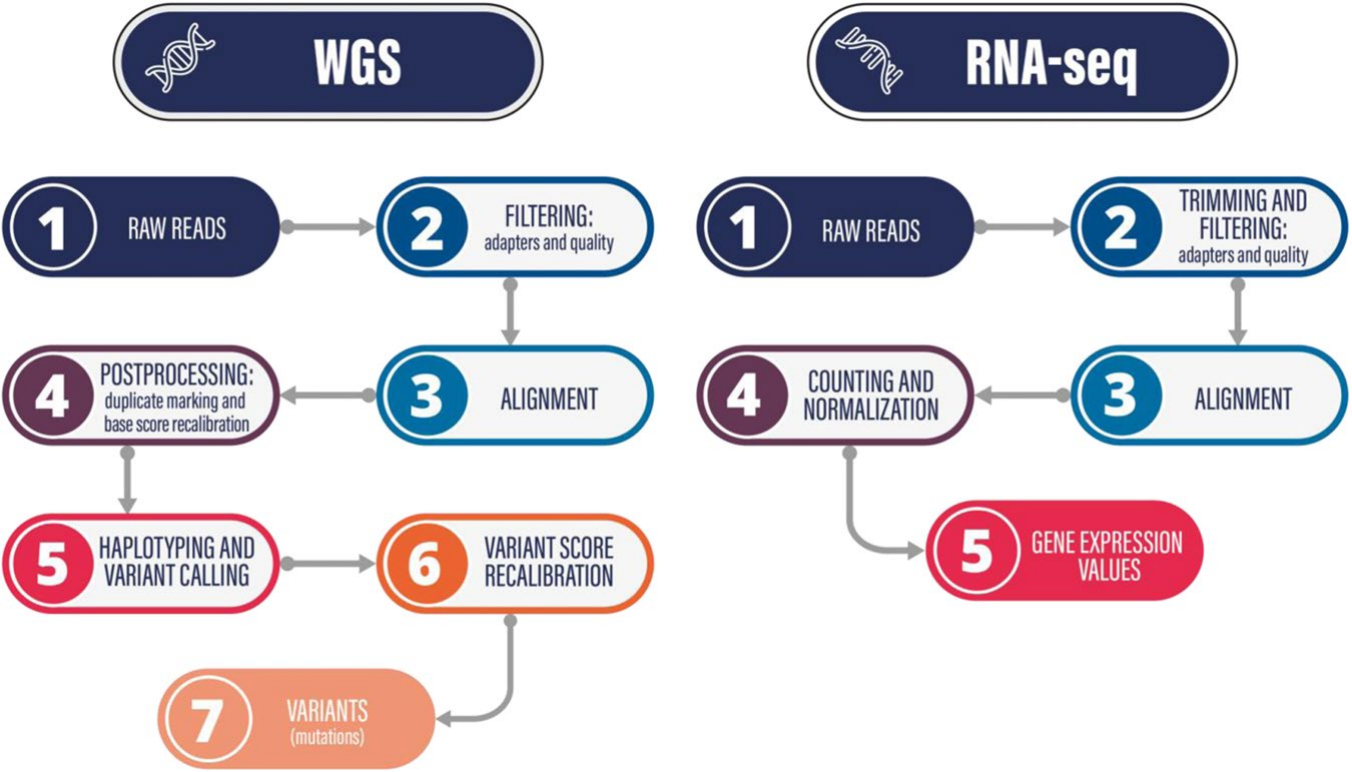
Data analysis pipelines for WGS and RNA-seq data.

The raw reads were filtered to remove low quality bases and artifacts such as adapter residues, according to the criteria of the sequencing provider. These criteria were to remove reads that contain adapters, reads that contain over 10% N basecalls (unidentified bases), and reads that contain low quality bases (Q score <  = 5) in greater quantity than 50% of the total read length. The filtered reads were aligned to the hg38 human genome with the mem algorithm of BWA^29^, using default settings and paired-end mode. GATK^30^ with Picard^31^ was used for postprocessing and variant calling. In the aligned reads the duplicates were marked, then we recalibrated the base quality scores using the two-step process from GATK and the known SNPs from the 1000 Genome Project^32^ and dbSNP^33^ (part of the GATK bundle). We generated individual VCF files by calling the haplotypes, then we combined them into a single file to perform joint genotyping. The alignments were rebuilt and scores were recalibrated around the variation sites, using the two-step variation recalibrator procedure of GATK for both SNPs and InDels to get the final VCF files with all types of variations and correct builds. An overview of the pipeline is presented in Fig. 2. The script for the WGS analysis pipeline is accessible as a GitLab project, where you can also find a detailed description on the project wiki page (please see the Code Availability section for the link).

### RNA-seq

#### Sample preparation and sequencing

Please see the Related content^1^.

#### Data analysis

The standard Illumina adapters were removed from the raw reads with TrimGalore/cutadapt^34^, and the sequencing quality was monitored with FastQC^35^. STAR^36^ was used for the paired-end alignment to the hg38 genome, considering the splice junctions registered in the hg38.gtf annotation file. The expression levels were determined by normalizing and counting the reads with RSEM^37^ Fig. 2 shows the main steps of the data analysis as a flowchart. The script for the RNA-seq analysis pipeline is accessible as a GitLab project, where you can also find a detailed description on the project wiki page (please see the Code Availability section for the link).

### Single cell RNA-Seq

#### Sample preparation and sequencing

To go deeper into transcriptomics and examine gene expression on a single cell level, we selected two of the LCL samples (GM22648 and GM22649), which showed the most variation in our previous experiments^1^, and isolated single cells for RNA-seq. Single cell separation and Illumina compatible sequencing libraries were done with 10X Chromium Controller using Chromium Next GEM Chip G Single Cell Kit and Chromium Next GEM Single Cell 3′ Kit v3.1 (10X Genomics) according to the manufacturer’s protocol. Briefly, after single cell separation, double stranded cDNA was generated and amplified by 12 PCR cycles. Library construction was continued with fragmentation, end repair and A-tailing, adaptor ligation steps and finished with 14 PCR cycles of amplification. Library quality and fragment size distribution were checked using DNA high sensitivity chip on BioAnalyzer 2100 (Agilent Technologies). Libraries were sequenced on NextSeq 500 (Illumina, San Diego, CA) sequencer.

### Data analysis

**Fig. 3:**
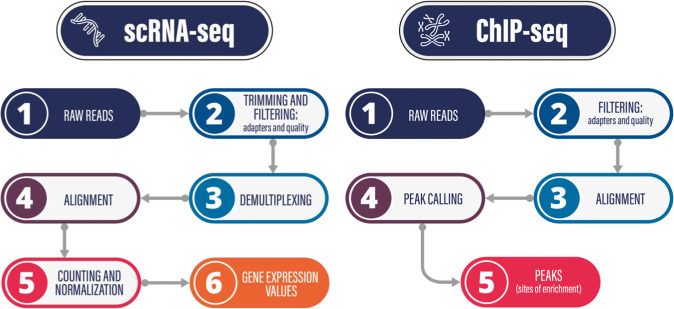
Data analysis pipelines for scRNA-seq and ChIP-seq data.

Cell Ranger (10X Genomics) was used for primary data analysis. Additional analysis/visualization was carried out with the Loupe Browser, a proprietary software of 10X Genomics. Sequencing data were demultiplexed with mkfastq and single cell feature counts were generated using cell ranger count commands. The overview of the analysis pipeline is shown in Fig. 3.

### ChIP-seq

#### Sample preparation and sequencing

Please see the Related content^1^.

#### Data analysis

As before, the standard Illumina adapters were removed from the raw reads with TrimGalore/cutadapt^34^, and the sequencing quality was monitored with FastQC^35^. The reads were aligned with the mem algorithm of BWA^29^ to the hg38 human genome, and samtools^38^ was used to generate sorted bam files. From the alignments, MACS2^39^ was used to call peaks with no input control. Figure 3 presents the schematic analysis pipeline. The script for the ChIP-seq analysis pipeline is accessible as a GitLab project, where you can also find a detailed description on the project wiki page (please see the Code Availability section for the link).

### Mass spectrometry

#### Sample preparation

Proteins were extracted from the cells using 80 µl lysis buffer (7 M urea, 30 mM Tris, 2 M thiourea, 4% CHAPS, pH 8.5). Samples were centrifuged at 1700g for 10 min at 4 °C and the collected supernatants were supplemented with 20 µl Laemmli buffer. 50 µl sample were loaded to 12% polyacrilamide gel and separated in a Bio-Rad mini tetra cell (Bio-Rad, cat. 1658004) on 100 V constant voltage for 1.5 hours. The protein bands were stained using PageBlue protein staining solution (Thermo Scientific, cat. 24620). Samples were divided into four fractions covering the whole column containing the separated proteins and each fraction was excised from the gel and further subjected to in-gel trypsin digestion. After destaining, the proteins were reduced using 20 mM dithiothreitol (Bio-Rad, cat. 1610611) for one hour at 56 °C followed by alkylation with 55 mM iodoacetamide (Bio-Rad, cat. 1632109) for 45 minutes in dark. Overnight trypsin digestion was carried out using MS grade stabilized TPCK-treated bovine trypsin (ABSciex, cat. 4445250) at 37 °C and the digested peptides were extracted and dried in SpeedVac (Thermo Scientific). The peptides were re-dissolved in 10 μl 1% formic acid before LC-MS/MS analysis. For LC-MS/MS analyses 5 µl samples were injected for each run and before the injection samples were supplemented with iRT peptides (Biognosys, cat. Ki-3002-1) according to the manufacturer’s instructions. The same fraction of the different samples was analysed in one batch file. All samples were analysed in duplicates.

#### LC-MS/MS analysis

Prior to mass spectrometry analysis, the peptides were separated using a 180 min water/acetonitrile gradient on an Easy 1200 nano UPLC system (Thermo Scientific). First, the peptide mixture was enriched on an Acclaim PepMap C18 (20 × 75 µm, 3 μm particle size, 100 Å pore size, Thermo Scientific, cat. 164946) trap column followed by a separation on an Acclaim PepMap RSLC (150 mm × 50 μm 2 μm particle size, 100 Å pore size, Thermo Scientific, cat. 164943) analytical column. The peptides were separated by a 5–7% gradient of solvent B over 5 minutes, 7–15% gradient of solvent B over 50 minutes, 15–35% gradient of solvent B over 60 minutes, 35–40% gradient of solvent B over 28 minutes and 40–85% gradient of solvent B over 5 minutes. After holding 85% solvent B for 10 minutes, the system returned to 5% solvent B in 1 minute followed by a 16-minute hold on. Buffer A was 0.1% formic acid in LC water (VWR International, cat. BDH23595.100) and buffer B was 0.1% formic acid in LC acetonitrile (VWR International, cat. BDH83639.100E). The flow rate was set to 300 nl/min.

Data-dependent acquisitions were performed on an Orbitrap Fusion mass spectrometer (Thermo Scientific) using Nanospray Flex ion source (Thermo Scientific). The spray voltage was set to static 2300 V with 2 Arb Sweep gas and the temperature of the ion transfer tube was set to 275 °C. Survey mass scans were performed in the Orbitrap analyser at 60000 resolution in 350–1600 m/z range in positive mode (AGC target: 4.0e5, RF lens: 60%, profile mode) followed by collision-induced dissociation tandem mass spectrometry of the 14 most intense ions in the ion trap analyser (AGC target 2.0e3, CID collision energy: 35%, CID activation time: 10 msec, Activation Q: 0.25, centroid mode). Precursor ions were selected by the peptide monoisotopic peak determination setting with a selection of ions with 2–7 charge states. Dynamic exclusion was set to place any selected *m/z* on an exclusion list for 45 seconds after a single MS/MS with +/− 10 ppm mass tolerance.

#### Data analysis of LC-MS/MS

**Fig. 4:**
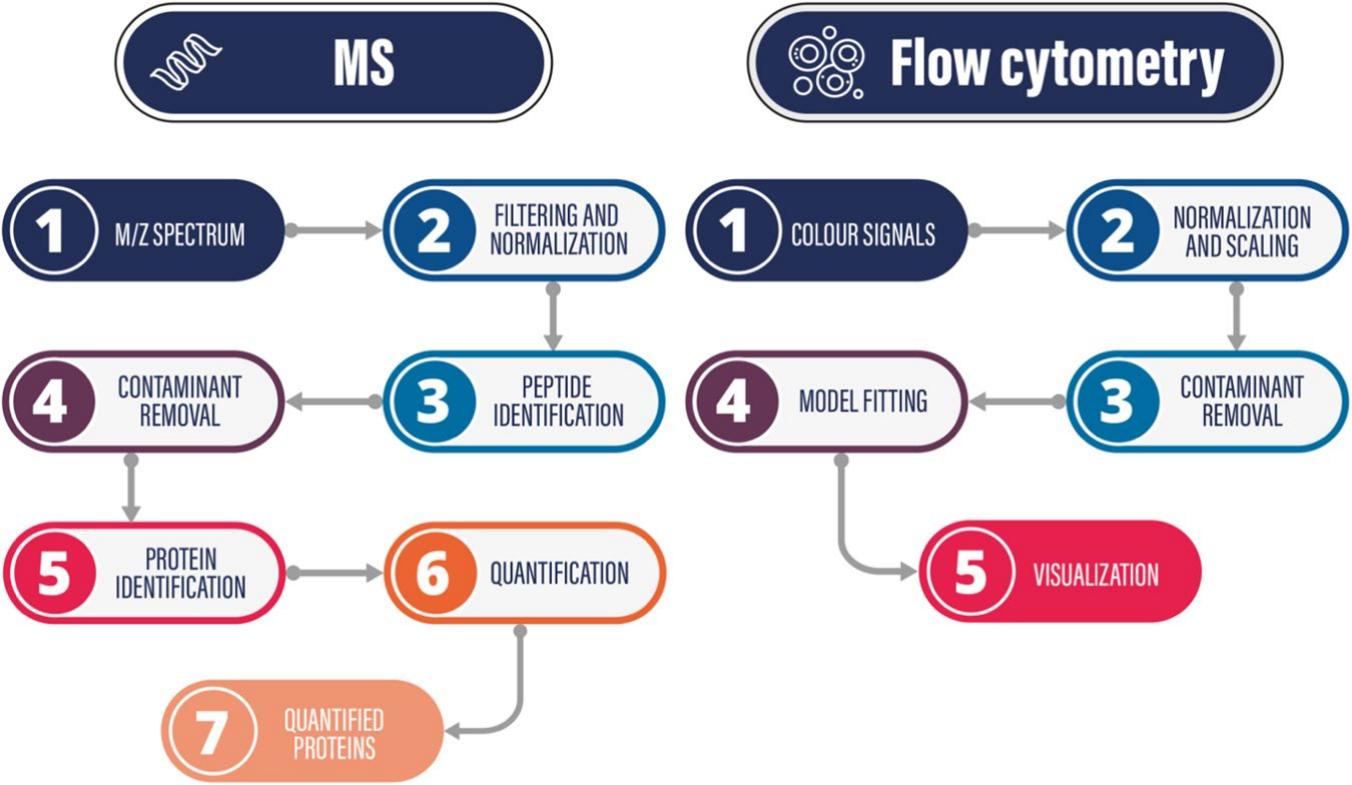
Data analysis pipelines for Mass Spectrometry (MS) and Flow Cytometry data.

LC-MS/MS spectra were searched against the human proteins downloaded from the UniProt database (release: 2018.10.10, 558590 sequence entries) and the sequence set of the iRT peptides using MaxQuant^40^ 1.6.2.10. search engine considering tryptic peptides up to two missed cleavages. Methionine oxidation, cysteine carbamidomethylation and N-terminal acetylation were considered as variable modifications. The recorded spectra were searched against the contaminant sequence database of the MaxQuant software as well. Proteins with minimum one identified peptide were accepted and in this step FDR correction was not applied. The results of the protein identifications were imported into the Scaffold 4.8.9 (Proteome Software Inc.) software. The four fractions of the same samples were combined and imported as one single file. Proteins were accepted as identification if minimum three peptides were identified at 0.1% FDR at the peptide level and 1% FDR at the protein level. For quantitative analysis, the ANOVA test was applied using total precursor intensity values after a Benjamini-Hochberg correction. The level of significance was set to p < 0.05. In Fig. 4 the steps of the data analysis are presented as a flowchart.

### Flow Cytometry

#### Sample preparation, measurements and data analysis

Please see the Related content^1^.

## Data Records

Table 1 summarizes the experiments done on each sample and it also shows the related databases and accession IDs to facilitate the easy access of the relevant data to a given experiment.

**Table 1:** Lookup table for all the experiments per sample, and their databases and identifiers to access the relevant datasets.

Sample	Analysis	Database	ID
GM22647	WGS	NCBI Sequence Read Archive	SRP266080^20^
	RNA-seq	NCBI Sequence Read Archive	SRP167344^21^
	ChIP-seq	NCBI Sequence Read Archive	SRP167344^21^
	MS	ProteomeXchange	PXD015169^25^
	FC	FlowRepository	FR-FCM-Z54Q^26^
GM22648	WGS	NCBI Sequence Read Archive	SRP266080^20^
	RNA-seq	NCBI Sequence Read Archive	SRP167344^21^
	scRNA-seq	NCBI Sequence Read Archive	SRP167344^21^
	ChIP-seq	NCBI Sequence Read Archive	SRP167344^21^
	MS	ProteomeXchange	PXD015169^25^
	FC	FlowRepository	FR-FCM-Z54Q^26^
GM22649	WGS	NCBI Sequence Read Archive	SRP266080^20^
	RNA-seq	NCBI Sequence Read Archive	SRP167344^21^
	scRNA-seq	NCBI Sequence Read Archive	SRP167344^21^
	ChIP-seq	NCBI Sequence Read Archive	SRP167344^21^
	MS	ProteomeXchange	PXD015169^25^
	FC	FlowRepository	FR-FCM-Z54Q^26^
GM22650	WGS	NCBI Sequence Read Archive	SRP266080^20^
	RNA-seq	NCBI Sequence Read Archive	SRP167344^21^
	ChIP-seq	NCBI Sequence Read Archive	SRP167344^21^
	MS	ProteomeXchange	PXD015169^25^
	FC	FlowRepository	FR-FCM-Z54Q^26^
GM22651	WGS	NCBI Sequence Read Archive	SRP266080^20^
	RNA-seq	NCBI Sequence Read Archive	SRP167344^21^
	ChIP-seq	NCBI Sequence Read Archive	SRP167344^21^
	MS	ProteomeXchange	PXD015169^25^
	FC	FlowRepository	FR-FCM-Z54Q^26^

The **WGS** datasets are available in the SRA database^20^, the raw sequencing files can be downloaded with the project ID SRP266080. The corresponding BioProject accession number is PRJNA627874. The comprehensive VCF file with all the detected variations is uploaded to Zenodo^22^ (10.5281/zenodo.6542293).

The **ChIP-Seq** datasets are also accessible from the SRA database^21^ under the project ID SRP167344 as raw reads, and the corresponding BioProject ID is PRJNA501889. The dataset and the project description can also be found in the GEO database^24^, the accession number is GSE121926.

The **RNA-Seq** datasets are bundled together with the ChIP-seq data, the raw FASTQ files of both can be found in the SRA database^21^ under the project ID SRP167344. The related joint BioProject is PRJNA501889. From the GEO database^24^ you can also access the data and their description with the experiment ID GSE121926 (again, together with the ChIP-seq data).

The **single cell RNA-seq** raw data is also uploaded to the same SRA project^21^ as the RNA-seq and ChIP-seq data: SRP167344. Likewise, the BioProject is common with those experiments, the accession number is PRJNA501889. With the ID GSE121926 the GEO record^24^ can be accessed, where links point to the scRNA-seq data (besides the ChIP-seq and bulk RNA-seq datasets). QC reports and comparative analysis reports created by the proprietary 10X Genomics software are available on Zenodo^23^ (10.5281/zenodo.6483461).

The **Proteomics** data is uploaded to ProteomeXchange^25^ (http://www.proteomexchange.org), it is accessible among the public datasets with the ID PXD015169 (10.6019/PXD015169).

The **Flow Cytometry** data is available at the Flow Repository^26^ (https://flowrepository.org) with the repository ID FR-FCM-Z54Q.

## Technical Validation

### Overview of experimental design

We compared five B-lymphoblastoid cell lines derived from the same individual, a batch of biological replicates. The goal was to identify the genomic variation in these supposedly stable cell lines and investigate their potential effect on the transcriptomics and proteomics level, as well as the chromatin structure: which genes have an altered regulation and expression, and how the affected protein levels change. We used a low passage number for the cell cultures to maintain genetic stability, as it was shown that up to 20 passages the genotypic discordance is negligible^41^. The cell culturing for our experiments started at passage number p8. Regarding ChIP Seq and RNA Seq experiments the total cumulative passage number was p14, while single cell experiments were performed at p18. These passage numbers included subculturing and seed stock preparations as well. See the experiment design overview in Fig. 1.

### Raw sequence quality control analyses

**Fig. 5:**
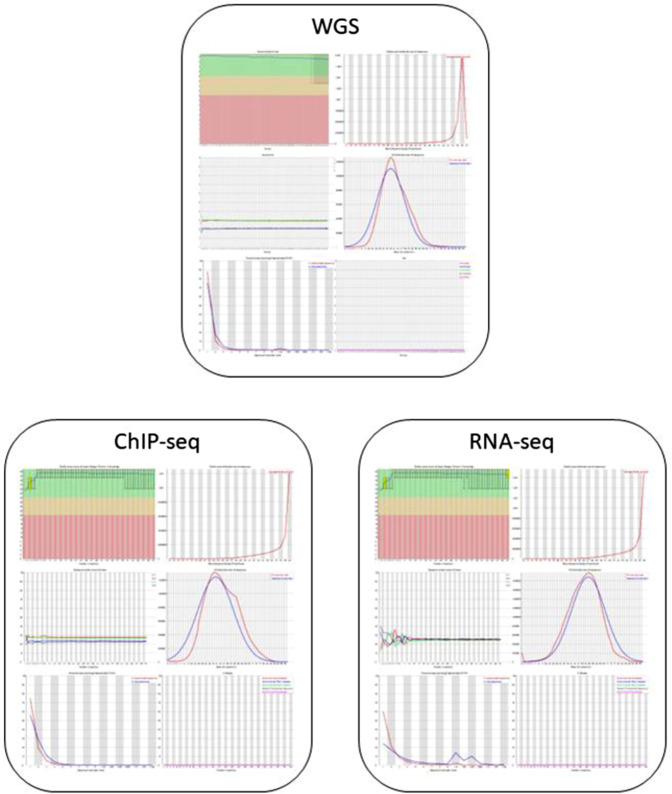
QC of representative Whole Genome Sequencing, ChIP-seq and RNA-seq raw datasets. The FastQC reports generally show high diversity and high base/read quality even before trimming, with expected peculiarities of the data types.

In every single case for the sequencing data, we checked the quality of the raw and trimmed sequences with FastQC. Figure 5 shows a representative example of FastQC results for WGS, ChIP-seq and RNA-seq data. None of the samples showed abnormalities derived from technical errors such as library preparation bias or sequencing failure, only some small deviations that are acceptable and characteristic of the data type. In general, the samples have high quality base callings (minimizing the chances of read errors), the expected GC content for the human genome/transcriptome, a high diversity of bases, and no sign of contamination, excessive adapter dimers or other artifacts. The RNA-seq data shows less diverse reads (more duplicates) and some polyT artefacts, but that is still acceptable, the transcriptome is less diverse than the genome, and polyT is used for capturing RNA during library preparation. Even before the trimming the adapter/barcode sequences in the reads are scarce, indicating an adequate fragment size distribution, and no oversequencing.

### Validation of biological features

As we stated previously, isogenic B-lymphoblastoid cell lines are highly stable genetically. We checked how similar they are by detecting all the mutations from the WGS data for the five cell lines and comparing them to each other. In Table 2 we collected the number of unique variations found in each cell line (those that are not occurring in any other of the five cell lines), showing the sequencing depth, mapping and coverage figures as well. We aimed for roughly 110–120 million reads per sample, and we got a very high (>99%) mapping ratio; at this depth we found that on average 9% of all the mutations (covered in all cell lines) are unique. Furthermore, this represents a 0.01% of all the possible sites on average in the human genome (its size was taken from the Ensembl database^42^). This proves that LCLs are indeed genetically very stable, the difference between them is smaller by an order of magnitude between average human genomes, which is reported to be 0.6%^32^.

**Table 2:** The differences between five isogenic LCLs.

	Average sequencing depth (PE)	Average mapping ratio	Average number of unique variants	Standard deviation of unique variants	Average coverage	Percentage of all the variants	Percentage of the human genome
GM22647	124,492,281	99.65%	443,481	58,810	5.87	9.43%	0.0143%
GM22648	110,198,283	99.61%	406,959	19,730	7.04	8.65%	0.0131%
GM22649	123,184,067	99.48%	376,305	3,481	7.02	8.00%	0.0122%
GM22650	117,120,399	99.68%	387,749	10,447	6.89	8.24%	0.0125%
GM22651	112,616,668	99.52%	429,532	18,043	6.56	9.13%	0.0139%

The first two columns show average sequencing depths and mapping ratios for the whole of the samples (not only the variants). The average numbers of unique variants are the variants that are found only in that given cell line (not common with the other cell lines); their standard deviations between replicates are shown in the next column. To the right there are the average coverages of the unique variants. The percentage of all the variants show the percent of the unique variants compared to all the variants that are found in the five cell lines (4,704,611). The percentage of the human genome is the number of unique variation sites compared to all the possible sites, basically the length of the hg38 genome (3,096,649,726).

**Fig. 6:**
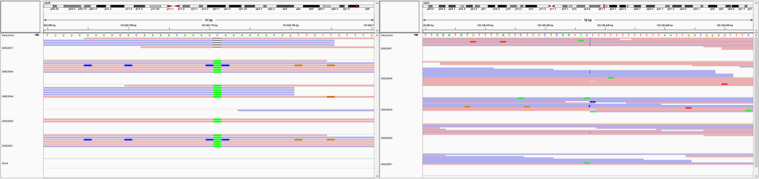
Genome browser screenshots of significant variations between the B-lymphoblastoid cell lines. In the centre of the left panel, a single base deletion in the GM22647 cell line is shown, whereas the other investigated cell lines have an adenine in the same position. In the centre of the right panel, an insertion in the GM22647 cell line is presented, whereas the reads in the other investigated cell lines show no insertion.

In spite of the high similarity, we managed to detect significant differences between the isogenic B-lymphoblastoid cell lines. A visual representation of the significant variations is shown in Fig. 6. Using the IGV genome browser^43^ we show the coverages on two sites where significant differences are detected: one of the cell lines exhibit a unique mutation. In the left panel (centre of the view) we see a single base deletion in the GM22647 cell line, while all the other cell lines have an A in that position (contrary to the C in the reference sequence). In the centre of the right panel there is a consistent insertion in the first cell line, while the reads in the other cell lines show no insertion at all, or an insignificant insertion. Even in the latter case, the inserted sequence (not visible) is different.

**Fig. 7:**
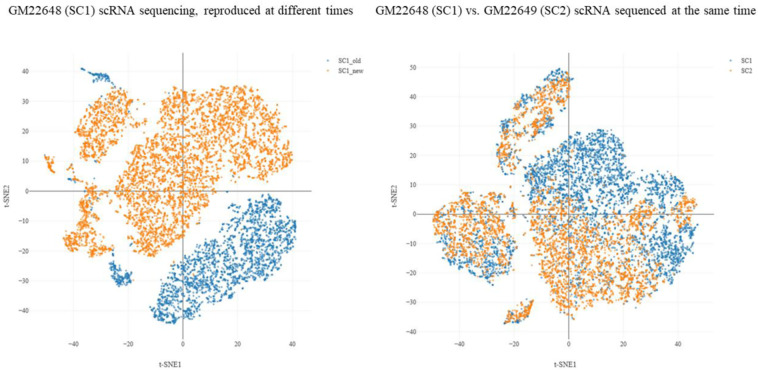
Comparison of single cell RNA-seq data by t-SNE analysis. The left panel shows the same cell line (GM22648, marked as SC1), samples were prepared and sequenced at different times (blue: earlier sequencing, orange: later sequencing). The batch effect is visible. The right panel represents two different cell lines (GM22648, SC1, and GM22649, SC2), prepared and sequenced simultaneously. Here we see partial overlaps, the differences are likely caused by actual expression differences, instead of batch effect.

We performed t-distributed stochastic neighbour embedding analysis (t-SNE) on the scRNA-seq data, based on the gene expressions we measured. We sequenced samples from two cell lines, GM22648 and GM22649. The GM22648 was prepared and sequenced twice, at two different timepoints. The GM22649 was processed and sequenced simultaneously with the second sequencing of GM22648. The results are shown in Fig. 7. When we compare the older and newer sequencings of GM22648, we see that the two samples prepared at different times form isolated groups. We consider it a batch effect, which is well known in single-cell sequencing^44^: samples prepared at different times, by different operators, with different reagents and more importantly sequenced to different depths can lead to widely different results. On the other hand, there is a much better overlap between the GM22648 and GM22649 experiments, performed at the same time. Thus we assume the remaining differences there are indeed valid biological differences between the two cell lines: the previously observed differences, such as variations in genomic sequence or histone marks, are translated to differential gene expressions. QC reports and comparative analysis reports created by the proprietary 10X Genomics software are available on Zenodo^23^ (10.5281/zenodo.6483461).

### Recommendations for downstream analyses

We processed **WGS** data to the level where we get alignment files, which contains the reads and their genomic coordinates where they map, and variant files, which contains the variants: alterations compared to the reference genome. These can be further processed e.g. by GATK^30^ or Picard^31^ to filter the variants, calculate statistics, and annotate the variants, deducing their effects in the genome. GATK offers a complete solution for every step of variant analysis, including comparing the discovered variants and genotypes to known controls, and Picard also has a lot of tools dedicated to working with genomic variations, such as vcf file manipulation tools and QC tools for WGS. With the list of genes that are altered, the next step is to find out their function, or the cellular component affected by their product, or the pathways and interactors they are related to. For these analyses one can use annotator, gene ontology and pathway analysis tools, including Reactome^45^, DAVID^46^ or STRING^47^. There are countless possibilities for further downstream analyses; the SequencEnG^48^ website provides an overview of the full WGS (and other NGS) data analysis process along with example software tools and pipelines.

Our pipeline for **ChIP-seq** data analysis yields mainly alignment files and peak files with the latter containing regions with significant enrichment. The visualization of the enrichment profiles and running basic statistical tests (e.g. plotting the enrichment levels of overlapping peaks against each other between two samples or calculating their correlation) can allow the better understanding of the sample variability. Genome-wide heatmaps of the enrichment, hierarchical clustering and Principal Component Analysis allows the identification of the similarities between the samples. R^49^ is recommended for such tests and visualizations as many statistical tests are available in the base package and its functionality can be extended by other packages, for example, ggplot2^50^ for visualizations. The alignments and peaks (and e.g. neighbouring genes with the right annotation track) can be viewed in genome browsers like the UCSC Genome Browser^51^. The enrichment profiles can be compared by groups: e.g. the R package DiffBind^52^ normalizes enrichment data, selects consensus peaks common between samples and groups, and identifies differential enrichments, where one group has a significantly different enrichment than the other. The differentially enriched peaks (or any other peak) can be assigned to certain genes to understand their functions, their roles in signalling pathways or diseases, or to see the affected cellular component. Software tools like Reactome^45^, DAVID^46^ and STRING^47^ can perform such annotation and ontology, as well as pathway analyses. One may also look for common motifs in the enriched regions, which are indicative of transcription factor binding sites usually. The Homer^53^ software suite includes tools for motif search. There are countless possibilities for further downstream analyses; the SequencEnG^48^ website provides an overview of the full ChIP-seq (and other NGS) data analysis process along with example software tools and pipelines.

The outputs of our **bulk RNA-seq** pipeline are primarily alignment files (to the transcriptome and the genome) and counts (expression levels on both gene level and transcript level). With this information one can already gain insight into the transcriptomic profiles of the samples, various standard statistical tests and visualization methods can be applied, e.g. the variance of gene expression can be calculated within a sample, or between samples for the same genes, or the correlation (covariation) of the expression of two genes across the samples. The counts per genes across various samples can be visualized in a heatmap, and hierarchical clustering can be performed to see the relation between the samples and so on. The similarity of two samples can be displayed as scatterplots, plotting the counts of the corresponding genes against each other. R^49^ is an ideal platform for such tests and visualizations, for many statistical tests functions are readily available in the base package, but one can always find and download packages for specialized tasks as well, e.g. ggplot2^50^ for visualization. Alignments in themselves or with annotations can be browsed with genome browsers like the UCSC Genome Browser^51^. The transcriptome profiles can be used to represent a group of samples and the groups can be compared (after normalization) to find differentially expressed genes. This is done, for example, by the R packages EdgeR^54^ or DESeq. 2^55^. Once you have the differentially expressed genes, you can identify what their functions are, and in what signalling pathways they participate, or what biological process, cellular component or disease they are related to. There are several databases and annotation, pathway and ontology software tools that can perform this type of analysis to reveal the higher meaning of your data. Some examples are Reactome^45^, DAVID^46^ and STRING^47^. There are countless possibilities for further downstream analyses; the SequencEnG^48^ website provides an overview of the full RNA-seq (and other NGS) data analysis process along with example software tools and pipelines.

As for **single cell RNA-seq**, the whole pipeline and the downstream analysis options are identical in many ways to the bulk RNA-seq analysis. One difference is that the library contains artificial sequences in the reads: molecular barcodes and unique molecular identifiers (UMIs); these are needed to track the cell of origin and reduce bias from the heavy PCR amplification. They must be taken into account at the trimming/alignment stages, but as they are library specific, the library kit manufacturer often provides proprietary tools and a strict guide that are suited for its specific construct. Although there are free tools that can handle scRNA-seq reads (e.g. STAR has a specialized version for scRNA-seq, STARsolo^56^, which you can adjust to your library construct), we chose to use the proprietary toolset from 10X Genomics, which included tools for further analyses as well, like expression profiling, batch correction and visualization. The other peculiarity of scRNA-seq datasets is that they are quite sensitive to batch effects, because the individual cells often come from different isolations, yielding different amount of RNAs, prepared by different operators, using different reagent lots and so on, therefore batch correction is highly recommended after counting^44,57^. Several free tools are available for this task, using various approaches (mutual nearest neighbors, deep learning, dimensionality reduction and others)^58^; some examples are Seurat^59^, Harmony^60^, iSMNN^61^, and DESC^62^. Apart from handling the specific library construct and correcting the batch effects, however, the same tools can be used as for bulk RNA-seq, as they are insensitive to whether the reads are coming from bulk RNA or single cell RNA. For example, the UCSC Genome Browser^51^ can be used for visualization, EdgeR^54^ and DESeq. 2^55^ can be used for differential expression analysis, and DAVID^46^ and STRING^47^ can be used for gene ontology and pathway analysis. In fact, bulk RNA tools like DESeq.2 and EdgeR proved to be quite popular in the single cell world too, many studies were conducted with them^63–67^, although more single cell oriented tools exist as well, like DEsingle^68^ and Monocle^69^. The SequenceENG^48^ website provides an overview of the full scRNA-seq (and other NGS) data analysis process as well along with example software tools and pipelines.

Regarding **proteomics**, we analysed the LC-MS/MS data with MaxQuant^40^, which yields identified proteins (or protein groups were they are ambiguous) and peptides, and quantitative information based on their intensity. There are several software tools that accept MaxQuant output files directly, such as MSstats^70^. This R package can handle several of the downstream analysis steps: it can check the quality of the datasets, remove contaminants and ambiguous proteins, perform normalization, and identify consensus proteins between groups of samples. After all the normalization, transformation and filtering steps, groups of samples can be compared to find proteins with different abundance. The protein levels can be visualized in various informative ways, including boxplots, heatmaps and volcano plots. The analysis of nucleic acid and protein data is not so different: once we have the list of differentially abundant proteins, we can also feed them to the previously mentioned software tools, like Reactome^45^, DAVID^46^ or STRING^47^, which can reveal various properties of the proteins and their genes through annotation, ontology and pathway analysis, like their functions, their role in the cell, what they are interacting with.

## Usage Notes

Figures 1–4 summarize the data analysis pipelines for further analysis.

## Data Availability

WGS: https://gitlab.com/smlce/unideb_wgs RNA-seq: https://gitlab.com/smlce/unideb_rna-seq ChIP-seq: https://gitlab.com/smlce/unideb_chip-seq
